# Respiratory microbiome and metabolome features associate disease severity and the need for doxycycline treatment in children with macrolide-resistant *Mycoplasma pneumoniae*-mediated pneumonia

**DOI:** 10.3389/fcimb.2025.1537182

**Published:** 2025-07-28

**Authors:** Wei-Chao Liao, Shiao-Wen Li, En-Wei Hsing, Sung-Han Hsiao, Ian Yi-Feng Chang, Yin-Cheng Chen, Yi-Yin Chen, Yi-Jiun Pan, Yu-Chia Hsieh

**Affiliations:** ^1^ Molecular Medicine Research Center, Chang Gung University, Taoyuan, Taiwan; ^2^ Department of Pediatrics, Chang Gung Children’s Hospital, Chang Gung Memorial Hospital, Chang Gung University, College of Medicine, Taoyuan, Taiwan; ^3^ Department of Life Sciences, National University of Kaohsiung, Kaohsiung, Taiwan; ^4^ Division of Infectious Diseases, Department of Medicine, Chang Gung Memorial Hospital, Taoyuan, Taiwan; ^5^ Institute of BioMedical Informatics, National Yang Ming Chiao Tung University, Taipei, Taiwan; ^6^ Graduate Institute of Immunology, College of Medicine, National Taiwan University, Taipei, Taiwan; ^7^ Department of Microbiology and Immunology, School of Medicine, College of Medicine, China Medical University, Taichung, Taiwan

**Keywords:** *Mycoplasma pneumoniae*, pneumonia, respiratory, microbiome, metabolome, macrolide-resistant *Mycoplasma pneumoniae*, disease severity

## Abstract

**Introduction:**

Commensal bacterial community along the upper respiratory tract functions against pathogens. The host determinants of *Mycoplasma pneumoniae* severity should be identified against the increasing threat of macrolide-resistant *M. pneumoniae* (MRMP) infection. We hypothesized that respiratory microbiome is involved in the clinical manifestations of *M. pneumoniae* infection.

**Methods:**

From 2017 to 2020, 92 children with MRMP-mediated pneumonia were enrolled among 845 children with community-associated pneumonia. Oropharyngeal samplings were collected within 48 h after admission. We compared respiratory microbiome and metabolites based on patients’ later development of prolonged fever and the need for doxycycline treatment (DT, *n* = 57) and the cured control without fever or doxycycline treatment (WDT, *n* = 35) by using 16S rRNA-based sequencing and untargeted metabolome analysis.

**Results:**

Significantly higher diversity and different respiratory microbiomes were evaluated in WDT patients in contrast to DT patients. *Fusobacterium*, *Haemophilus*, *Gemella*, *Oribacterium*, *Actinomyces lingnae*, *Fusobacterium periodonticum*, *Gemella sanguinis*, and *Solobacterium moorei* were inversely correlated with disease severity. We assumed that metabolites of divergent microbiomes were related to MRMP development. We identified 15 discriminative amino-acid- and fatty-acid-related metabolites in two groups. *F. periodonticum* abundance was negatively associated with an inflammatory metabolite: a platelet-activating factor. *Fusobacterium* and *Oribacterium* were related to the decrease in LysoPE(18:1(9Z)/0:0) and LysoPC(18:1(9Z)).

**Conclusions:**

Microbiota dysbiosis with dysregulated inflammatory glycerolphospholipid-related metabolites was related to disease severity and the need for doxycycline treatment in children with MRMP-mediated pneumonia. Anaerobic bacteria metabolites and metabolic pathway could be beneficial therapeutic targets against *M. pneumoniae* infection.

## Introduction

*Mycoplasma pneumoniae* is a common pathogen that causes respiratory tract infection in children, which can range from mild pharyngitis to life-threatening acute respiratory distress syndrome (Waites and Talkington, 2004). Several studies have reported that *M. pneumoniae* can trigger and exacerbate asthma and contributes to difficulties with asthma management (MacDowell and Bacharier, 2005). Even without preceding respiratory symptoms, it may be associated with numerous extrapulmonary manifestations mediated by direct invasion or immune-mediated inflammation, including vasculitis, thrombosis, hepatitis, Stevens–Johnson syndrome, encephalitis, encephalopathy, and Guillain–Barré syndrome (Waites and Talkington, 2004).

After comprehensive pneumococcal conjugate vaccine implementation, *M. pneumoniae* is still detected in up to 19% of hospitalized children aged ≥5 years with community-acquired pneumonia (Kutty et al., 2019). Notably, 12% of children are admitted to the intensive care unit (ICU) (Kutty et al., 2019). Along with the overuse of macrolides as the empirical treatment in individuals suspected with *M. pneumoniae* infection, the prevalence rate of macrolide-resistant *M. pneumoniae* (MRMP) in Asians is up to 90%–100% (Hung et al., 2021). Patients with MRMP infection are usually associated with a longer duration of fever and hospitalization (Hung et al., 2021). In a study conducted in Ohio, USA, despite the low MRMP prevalence (2.8%), MRMP-infected patients have an approximately fivefold higher risk of ICU admission than those infected with macrolide-susceptible strains (Lanata et al., 2021). National outbreak of MRMP infection has been reported in children and adults in Japan, Korea, and Taiwan (Lee et al., 2018; Hung et al., 2021).

In patients with MRMP infection, quinolone and minocycline/doxycycline are effective antimicrobial agents. In particular, minocycline/doxycycline can rapidly decrease the amount of MRMP in the nasopharynx (Okada et al., 2012). Although macrolide cannot kill MRMP, some patients are clinically responsive to macrolide, possibly due to its immunomodulatory effect and the self-limiting property of *M. pneumoniae* infection (Suzuki et al., 2006). Usually, medication would shift to quinolone or doxycycline after 3 days of macrolide treatment in patients with MRMP pneumonia and no sign of defervescence. This is because persistent inflammation may have induced respiratory difficulty and worsen health conditions. Because of the extensive clinical syndrome caused by *M. pneumoniae* infection and the increasing threat of macrolide resistance, the host determinants of *M. pneumoniae* severity should be identified to develop complementary strategies against *M. pneumoniae* infection.

During 2017 to 2019, Taiwan experienced a community outbreak of MRMP infection (Hung et al., 2021). When children were diagnosed with community-acquired pneumonia, augmentin and/or azithromycin were the most commonly used antimicrobial agents. If prolonged fever occurred after suspected MRMP infection, doxycycline was applied after azithromycin usage for 3 days. In a previous study, respiratory commensal microbiotas modified disease susceptibility to acute respiratory disease (Abt et al., 2012). In this study, we investigated whether the respiratory microbiota at the beginning of hospitalization was associated with disease course and response to antibiotic treatment in children with MRMP-mediated pneumonia. We also analyzed the respiratory untargeted metabolomics for insights into the pathophysiological significance of MRMP pneumonia.

## Methods

### Patient sample collection and clinical assessment

**Figure 1:**
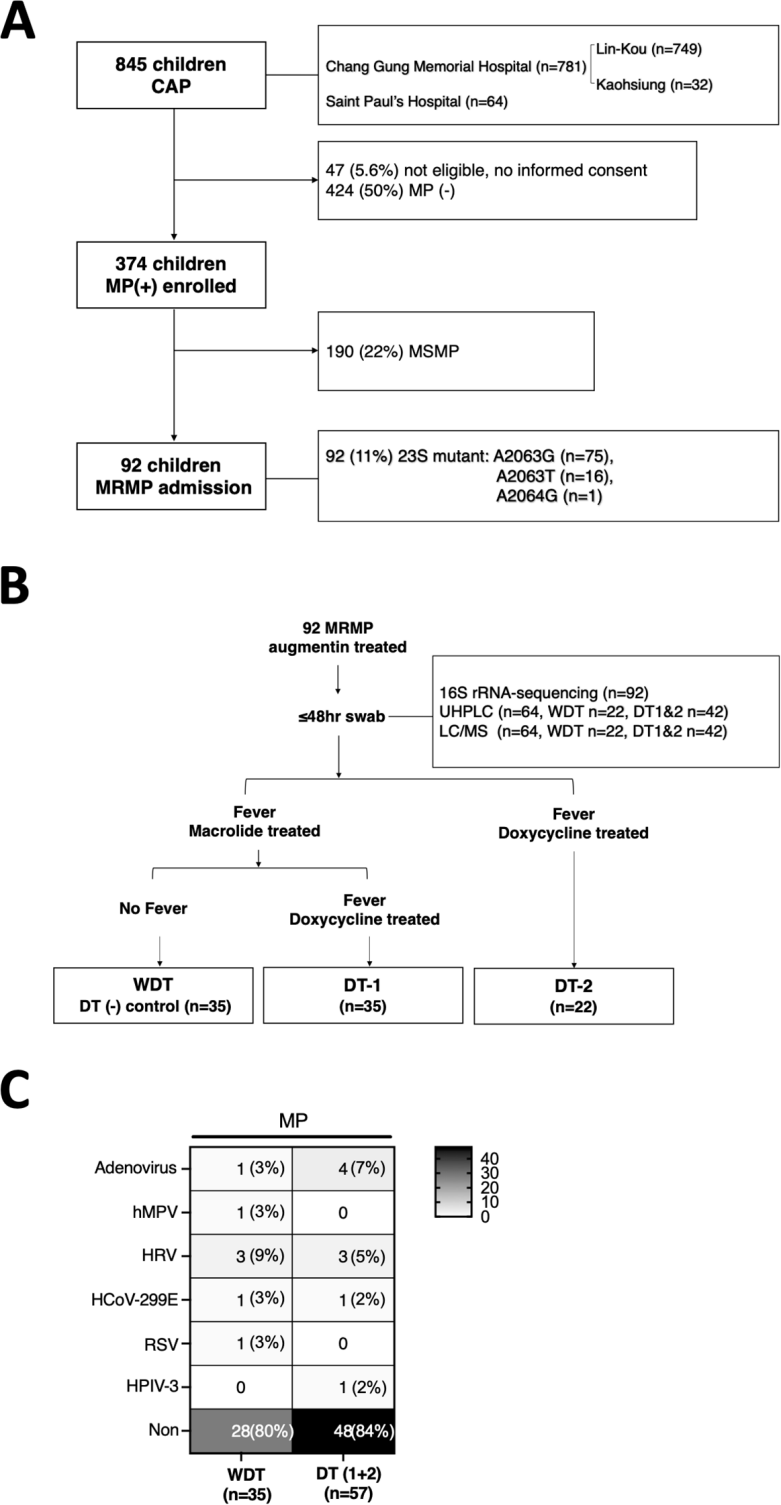
Scheme of sample collection and analyses. **(A)** Throat swabs of 845 patients were uniformly collected within 48 h after admission at CGMH: Lin-Kou & Kaohsiung branch (LCGMH) and Saint Paul’s Hospital from April 2017 to March 2020. A total of 92 patients with MRMP were included in this study and were categorized. **(B)** Patients with MRMP without doxycycline treatment and no persistent fever were classified as the cured control, WDT (*n* = 35). Patients with MRMP who needed doxycycline treatment (DT) and with persistent fever were classified as DT-1 (*n* = 35) and DT-2 (*n* = 22). **(C)** Among them, over 80% of patients were infected by MRMP alone without other pathogens detected.

We prospectively enrolled 845 children aged <18 years, admitted to Chang Gung Memorial Hospital: Lin-Kou and Kaohsiung (LCGMH) branch and Saint Paul’s Hospital from April 2017 to March 2020, and diagnosed with community-acquired pneumonia (**Figure 1A**). After written informed consent was obtained from patients or their parents, throat swabs (FLOQSwabs, Copa; Murrieta, CA, USA) were collected from the patients by pediatricians within 48 h after admission. *M. pneumoniae* pneumonia was diagnosed based on clinical symptoms, chest radiography, and real-time polymerase chain reaction (PCR), and assessed based on macrolide resistance to 23S rRNA gene of bacteria with conventional PCR from *M. pneumoniae*-positive case specimens (Hung et al., 2021). FilmArray respiratory panel 2.1 (BioFire^®^, USA) was used to detect whether patients were co-infected by other pathogens, besides MP (**Figure 1C**).

A total of 92 patients with MRMP pneumonia were enrolled. In the analysis of mutations at 23S rRNA of bacteria, 81.5% (75/92) of mutants at A2063G, 17.4% (16/92) of mutants at A2063T, and 1% (1/92) of mutants at A2064G were assessed. Augmentin with or without azithromycin was used as the antimicrobial agent in 92 patients. If patients with suspected MRMP had persistent fever after 3 days of treatment of azithromycin, they would be treated with doxycycline instead (**Figure 1B**). Some patients were initially treated with doxycycline with or without augmentin after admission, which was decided by each primary care physician based on the patients’ condition and personal experience. Patients were included in this study with no underlying diseases and if they meet the following conditions: (1) Before admission, patients were treated with medication other than augmentin/azithromycin; (2) after admission, patients were treated with medication other than augmentin/azithromycin/doxycycline. Medical information and laboratory results were collected. Pneumonia severity was evaluated by the standard scoring system as follows: (1) Fever for >10 days (1 point); (2) <93% oxygen saturation measured by pulse oximetry (1 point); (3) chest x-ray finding of the presence of the following: (A) unilobar consolidation (1 point), (B) multilobar consolidation (2 points), (C) unilateral pleural effusion (1 point), and (D) bilateral pleural effusion (2 points); and (4) C-reactive protein level: (A) 50–100 mg/L (1 point), (B) 101–200 mg/L (2 points), (C) 201–300 mg/L (3 points), and (D) >400 mg/L (4 points).

### 16S rRNA-targeted library preparation and sequencing

We sequenced the 16S rRNA gene from throat swab specimens and used the 16S rRNA gene library via an Illumina MiSeq platform to generate paired 300-bp reads. Detailed information is presented in the supplementary appendix.

### 16S metagenomic bioinformatics analysis

16S rRNA gene sequences were processed to classify microbial constituents using the DADA2 pipeline as in a previous study (Callahan et al., 2016). The truncated forward and reverse sequences were defined at positions 290 and 220, respectively, and the first 13 bases of each sequence were trimmed. Total sequences were used to construct amplicon sequence variants (ASVs), and ASVs comprising <10 reads in at least two samples were filtered from the dataset. A Naïve Bayes classifier was trained by using the most recent available version of Silva (release 138.1) sequences for taxonomy assignment.

### Microbial community analysis

Children with MRMP infection were categorized into two groups: the control group [without doxycylcine treatment (WDT)] and the doxycycline treatment (DT) group. The DT group was further divided into two groups: those suffering from prolonged fever and those who were doxycycline treated (**Figure 1B**). The differences in microbial composition between groups were examined by using microbial community analysis. Alpha-diversity was calculated based on species richness and evenness by using several metrics including observed species ASVs, Chao1 index, and Shannon index with phyloseq as in a previous study (McMurdie and Holmes, 2013). The contribution of covariates towards differences in the microbial profile of all samples was computed by using the Kruskal–Wallis test. Beta-diversity was calculated to assess the similarity of communities between groups using principal coordinate analysis (PCoA) with the unweighted UniFrac distance as in a previous study (Lozupone and Knight, 2005) [*p* < 0.05 by permutational multivariate analysis of variance (PERMANOVA) test]. Statistically significant taxa for each group were evaluated by the linear discriminant analysis (LDA) of effect size (LEfSe) analysis, which employed the non-parametric factorial Kruskal–Wallis test, Wilcoxon rank sum test, and LDA to identify the differentially abundant biomarkers between two groups (LDA >3 and *p* < 0.05) as in a previous study (Segata et al., 2011).

### Co-occurrence network and statistical network estimation

The microbial association network was estimated by using NetCoMi to compare microbial interactions that are likely to change between conditions as in a previous study (Peschel et al., 2021). The Jaccard index including several measures (degree, betweenness centrality, closeness centrality, and eigenvector centrality) was used to assess the dissimilarities between the two networks. The adjusted Rand index (ARI) was implemented to test similarities between two clusters.

### Untargeted metabolomic profiling in human throat (respiratory tract)

Untargeted metabolomics were acquired from throat swabs of 64 (70%) willing participants (**Figure 1B**). Ten microliters of samples dissolved in extract solution (acetonitrile:methanol:water = 2:2:1) was injected into the vanquish focused ultrahigh-performance liquid chromatography (UHPLC) system coupled with Orbitrap Elite Mass Spectrometry (Thermo Fisher Scientific, USA) by using electrospray ionization. Profiling using the liquid chromatography–tandem mass spectrometry (LC-MS/MS) platform was carried out by BIOTOOLS CO., LTD (Taiwan).

### Metabolomics data pre-processing and analysis

Raw data files were converted into the mzML format by using ProteoWizard and processed by using the R package XCMS (version 3.2). Pre-processed data were normalized by the sum of total peak area, log transformed, and autoscaled (mean-centered and divided by the standard deviation of each variable) prior to downstream statistical analysis. For multivariate statistical analysis, orthogonal projection to latent structures-discriminant analysis (OPLS-DA) was performed by using Metaboanalyst 5.0 as in a previous study (Pang et al., 2021). The predictive performance of the PLS-DA model was assessed by using the cumulative Q2Y metric, which had values between 0 and 1. High Q2Y indicated excellent performance. Differential metabolites were filtered by using the variable importance in projection (VIP) score >1 generated from the OPLS model and the *p-*value obtained from Student’s *t*-test. The metabolites were statistically significant if VIP ≥1.5 and *p* < 0.05. For univariate analysis, significant metabolites differentially regulated in the two groups were analyzed with volcano plots that included |log_2_FC| > 1 and *p* < 0.05 via Student’s *t*-test.

### Correlation of bacteria ASVs and metabolite abundance

Pairwise Spearman’s rank correlation was computed among the 12 genera (10 species) and 15 metabolites differentially abundant between groups. Finally, we examined how MRMP-associated metabolites were related to MRMP-associated bacterial taxa using Spearman’s correlation. Only correlations with |Spearman’s *r*| >0.4 and FDR ≤ 0.001 were used for cluster analysis.

### Statistical analysis

The median values and interquartile ranges (IQRs) were calculated for continuous variables, and percentages were used for categorical variables. For continuous variables, comparison of means was conducted by using unpaired *t*-test. For categorical variables, chi-square test or Fisher’s exact test was used. *p* < 0.05 was considered statistically significant. All statistical analyses were performed using SPSS 22.0 for Windows (Statistical Package for Social Sciences).

## Results

### Study population and enrollment

Ninety-two MRMP-mediated pneumonia specimens were collected among 845 patients without doxycycline treatment before admission (**Figure 1A**). Briefly, 70 children with MRMP-mediated pneumonia were initially treated with augmentin and/or macrolide after admission. Among them, children achieving defervescence without doxycycline treatment (control group) were labeled as the WDT group (*n* = 35), and the other children treated with doxycycline 3 days later due to persistent fever (*n* = 35) were labeled as doxycycline treatment group-1 (DT-1). Another group of children with MRMP-mediated pneumonia with doxycycline +/− augmentin after admission (*n* = 22) were labeled as doxycycline treatment group-2 (DT-2) (**Figure 1B**; **Table 1**).

**Table 1:** Clinical characteristics of patients.

	WDT (*n* = 35)	DT (*n* = 57)		*p*-value		
		DT-1 (*n* = 35)	DT-2 (*n* = 22)	WDT vs. DT-1	DT-1 vs. DT-2	WDT vs, DT-1 and DT-2
Age, years	7.6	6.9	6.9	0.6	1	0.6
	(5.3–8.8)	(5.8–10.2)	(5.5–9.2)			
Sex, male	17 (48.6)	9 (25.7)	7 (31.8)	0.05	0.8	0.07
Fever days before hospitalization	6.0 (4–7)	5 (3.75–6.25)	6 (5–7)	0.4	0.06	0.9
Antibiotic use before hospitalization						
Augmentin	22 (62.9)	22 (62.9)	14 (63.6)	1	1	1
Azithromycin	21 (60)	20 (57.1)	18 (81.8)	0.8	0.05	0.7
Previous antibiotic						
**usage, days**	1 (0–3)	2 (0–3)	3 (3–5)	0.4	0.006^**^	0.03*
**Total fever days**	7 (6–8)	8 (6–10)	8 (6.75–10.5)	0.001^**^	0.7	<0.001***
Total						
**hospitalization days**	4 (3–5)	5 (4–6)	4 (3.5–6.0)	0.009^**^	0.7	0.008**
Ct value at diagnosis	29.1 (26.5–31.8)	28.8 (26.7–30.8)	28.5 (26.3–32.1)	0.38	0.6	0.4
Hypoxemia	0 (0)	3 (8.6)	3 (13.6)	0.24	0.7	0.08
Laboratory characteristic						
White blood cells (×10^9^/mL)	6,800 (5,807–9,050)	7,340 (6,247–10,075)	6,900 (5,850–8,800)	0.76	0.7	0.9
CRP content (mg/L)	15.9 (10–51.9)	30.8 (11.4–61)	27.2 (14.9–103.5)	0.28	0.3	0.1
Chest x-ray pattern						
Patchy consolidation	24 (68.6)	27 (77.1)	16 (72.7)	0.4	0.8	0.6
Pleural effusion	5 (14.3)	7 (20)	7 (31.8)	0.5	0.4	0.3
**Severity score**	1 (1–2)	2 (1–4)	2 (2–4.25)	0.01*	0.4	0.002**
**CRP score**	0 (0–0)	0 (0–1)	0.5 (0–1.25)	0.2	0.2	0.04*
Chest x-ray score	1 (0–1)	1 (1–1)	1 (1–1.25)	0.09	1	0.06

CRP, C-reactive protein; WDT, without doxycycline treatment; DT-1, doxycycline treatment 1 (patients treated with doxycycline 3 days after augmentin and/or macrolide treatment); DT-2, doxycycline treatment 2 (patients initially treated with doxycycline and/or augmentin after hospitalization). Data are given as median (interquartile range) or *n* (%). Statistically significant *p*-values were represented in asterisks. ***p < 0.001, **p < 0.001.

Across these three groups, age, bacterial load (Ct value) at diagnosis, white blood cell (WBC) counts, C-reactive protein (CRP), and chest x-ray pattern were not significantly different (**Table 1**). Moreover, fever days before hospitalization, previous augmentin usage, previous azithromycin usage, and previous antibiotic usage (days) before hospitalization were similar between WDT and DT-1 patients (*p* = 0.4). However, DT-2 patients had a longer antibiotic usage (days) before admission (*p* = 0.006), a higher rate of previous azithromycin usage before hospitalization (*p* = 0.05), and a tendency of longer febrile days before hospitalization (*p* = 0.06) than DT-1 patients. DT-1 and DT-2 patients had significantly longer febrile days, total hospitalization days, higher CRP score (*p* = 0.04), and higher severity score (*p* = 0.002) than WDT patients (**Table 1**). MRMP infection-only cases account for 80% (28/35) of WDT patients and 84% (48/57) of DT patients (**Figure 1C**). Since these 92 patients had MRMP leading to pneumonia with less initially exaggerated immune response (similar WBC expressions among groups), we proposed that a dysregulated microenvironment might be the main reason for the prolonged antibiotic usage and febrile days. We examined the microbiome to evaluate whether the community of microorganisms would be different among the three groups.

### Respiratory microbiota in patients with MRMP

**Figure 2:**
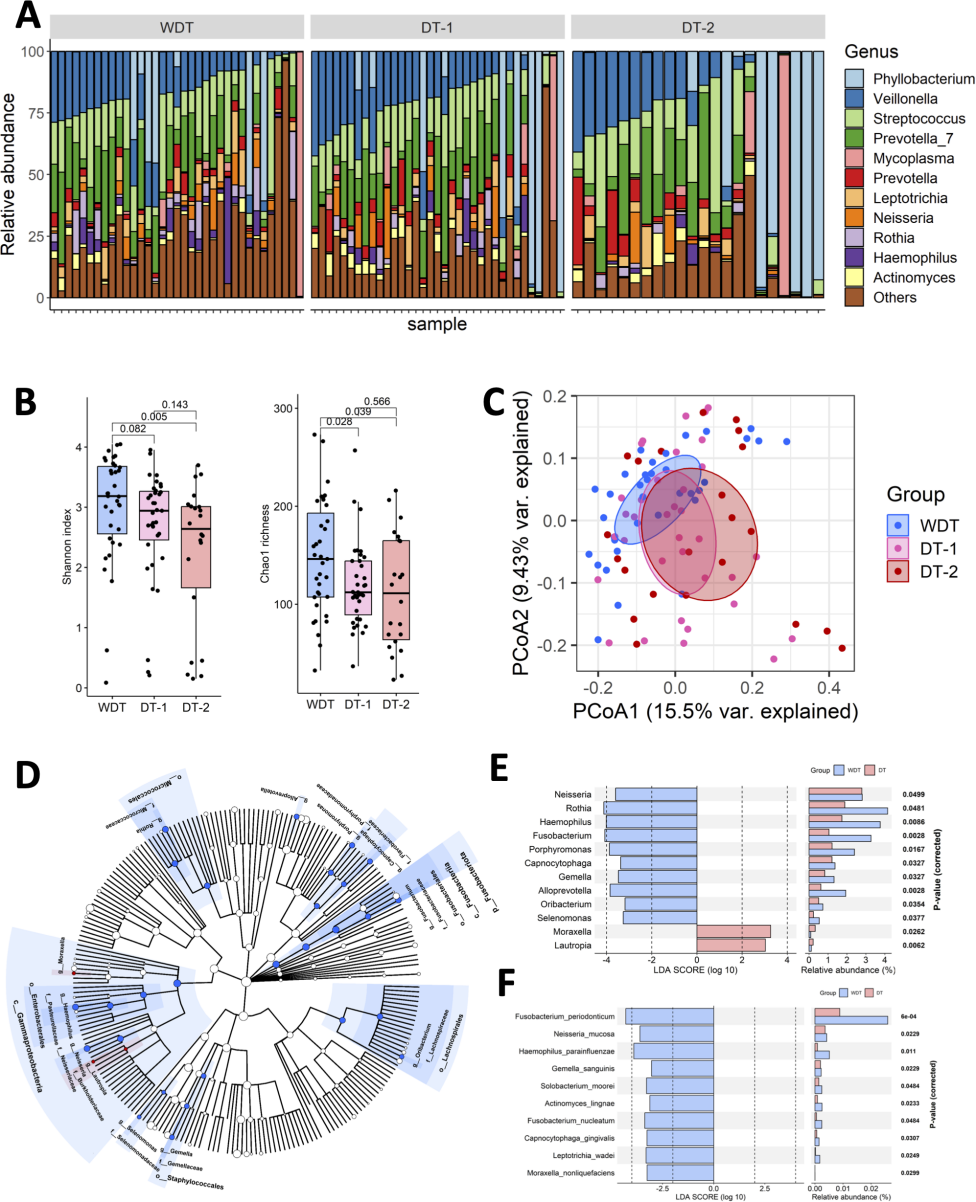
The respiratory microbial diversity in WDT was higher than the DT one. **(A)** Fraction of predominant microbiota with at least 2% relative abundance at the genus level in all samples. **(B)** Microbial richness based on the different alpha-diversity index in WDT (blue), DT-1 (pink), and DT-2 (red) patients. The box represented the interquartile range (IQR) and the midline represented the median. *p < 0.05. **(C)** Principal coordinates analysis (PCoA) for WDT (blue), DT-1 (pink), and DT-2 (red) patients with MRMP by Unweighted UniFrac distance. Significant differences were observed between WDT and DT patients. A PERMANOVA (α = 0.05) with 999 random permutations was run to determine differences between groups with the Vegan package. The composition of respiratory microbiome in WDT patients differed from those with DT, by linear discriminant analysis of effect size (LEfSE) analysis. **(D)** Circular taxonomic and phylogenetic trees of microbiota diversity. The relative color represented the more abundant bacterial taxonomy in each group. LDA scores revealed differentially abundant bacteria in the respiratory microbiomes in WDT (blue) and DT (red) patients at the **(E)** genus and **(F)** species levels.

A total of 92 throat samples were enrolled for 16S rRNA sequencing analysis to characterize respiratory microbiota profiles using the DADA2 pipeline and 8,513,410 qualified sequences (mean ± SD: 92,537.1 ± 22,870.2); 2,831 ASVs were obtained. After taxonomic assignment, the ASVs were identified as known taxa (11 phyla, 16 classes, 41 orders, 85 families, 164 genera, and 143 species) and unclassified groups. The bacteria taxonomic profiles at the genus level in the respiratory microbiota from our cohort were calculated, revealing the most abundant bacterial genera. The predominant bacterial composition in respiratory samples (approximately >2% sequences) included *Veillonella* (15.8%), *Phyllobacterium* (14.3%), *Prevotella*_7 (14.4%), *Streptococcus* (14.3%), *Mycoplasma* (4.2%), *Prevotella* (3.9%), *Leptotrichia* (3.4%), *Rothia* (2.8%), *Neisseria* (2.8%), *Haemophilius* (2.5%), and *Actinomyces* (2.2%), comprising 76.5%, 79.6%, and 85.8% of the respiratory microbiota in the WDT, DT-1, and DT-2 patients, respectively (**Figure 2A**).

### Respiratory microbiota landscapes among WDT, DT-1, and DT-2 patients

Although fever days and antibiotic usage days before hospitalization of WDT and DT-1 patients were similar, taxonomic profiling found that the microbial richness and diversity of WDT patients were higher based on the results of the Chao index (*p* = 0.028) than those in DT-1 patients, and the Shannon index (*p* = 0.082) indicated that the microbial evenness of DT-1 patients tend to be lower than those in WDT patients (**Figure 2B**). Despite DT-2 patients having longer fever days and antibiotic usage days before hospitalization than DT-1 patients, their microbial richness via the Chao index (*p* = 0.566) and diversity using the Shannon index (*p* = 0.143) were comparable. Microbial richness (*p* = 0.00602) and diversity (*p* = 0.0105) in WDT patients were significantly higher than those in DT patients (DT-1 + DT-2) (**Supplementary Figure S1**). Moreover, the between-sample diversity comparison carried out using PCoA based on unweighted UniFrac distances showed that the community composition of WDT patients was clustered distinctively from those of DT-1 and DT-2 patients (*p* < 0.01) (**Figure 2C**). Therefore, we combined DT-1 and DT-2 patients together as one DT patient group in the following evaluations.

### Composition differences between WDT and DT patients

High-dimensional class comparisons using LEfSe were performed to identify differentially abundant taxa. The results showed that 10 genera, *Neisseria, Rothia, Haemophilus, Fusobacterium, Porphyromonas, Capnocytophaga, Gemella, Alloprevotella, Oribacterium*, and *Selenomonas*, were enriched in WDT patients (**Figure 2D**), while 2 genera, *Moraxella* and *Lautropia*, were enriched in DT patients (**Figure 2E**). Moreover, 10 species, namely, *Fusobacterium periodonticum*, *Neisseria mucosa*, *Haemophilus parainfluenzae*, *Gemella sanguinis*, *Solobacterium moorei*, *Actinomyces lingnae*, *Fusobacterium nucleatum*, *Capnocytophaga gingivalis*, *Leptotrichia wadei*, and *Moraxella nonliquefaciens*, were enriched in WDT patients (**Figure 2F**). Interestingly, no species were enriched in DT patients.

### Comparison of microbial association networks

Microbial co-occurrence networks were constructed by NetCoMi to further assess the microbiota community systematically. Visual inspection revealed a global change of network topology based on disease courses and responses to antibiotic treatment in children with MRMP-mediated pneumonia (**Supplementary Figure S2**). The Jaccard index was used as a local network centrality (degree, betweenness centrality, closeness centrality, eigenvector centrality, and hub taxa) measure to evaluate the differences and similarities between our two networks. All measurements were lower than expected by random. Our results indicated completely different sets of central nodes between these networks. The ARI was 0.011 (*p* = 0.01), indicating a low similarity between the two clusters (**Table 2**). Both indexes showed that the groups of taxa or the overall network topologies were different between networks. Our investigation suggested that a major shift within the community structures was associated with disease severity and the need for doxycycline treatment.

**Table 2:** Jaccard index of co-occurrence networks.

	Jaccard index	*P*(≤Jacc)	*P*(≥Jacc)
**Degree**	0.212	0.000573***	0.999706
**Betweenness centrality**	0.08	0.000000***	1.000000
**Closeness centrality**	0.233	0.003477**	0.997991
**Eigenvector centrality**	0.264	0.048132*	0.967853
Hub taxa	0	0.197531	1.000000

Significant *p*-values were represented in asterisks. ***p < 0.001, **0.001 ≤ *p* < 0.01, and *p < 0.05.

### Respiratory microbial compositions were correlated with febrile days and disease severity

**Figure 3:**
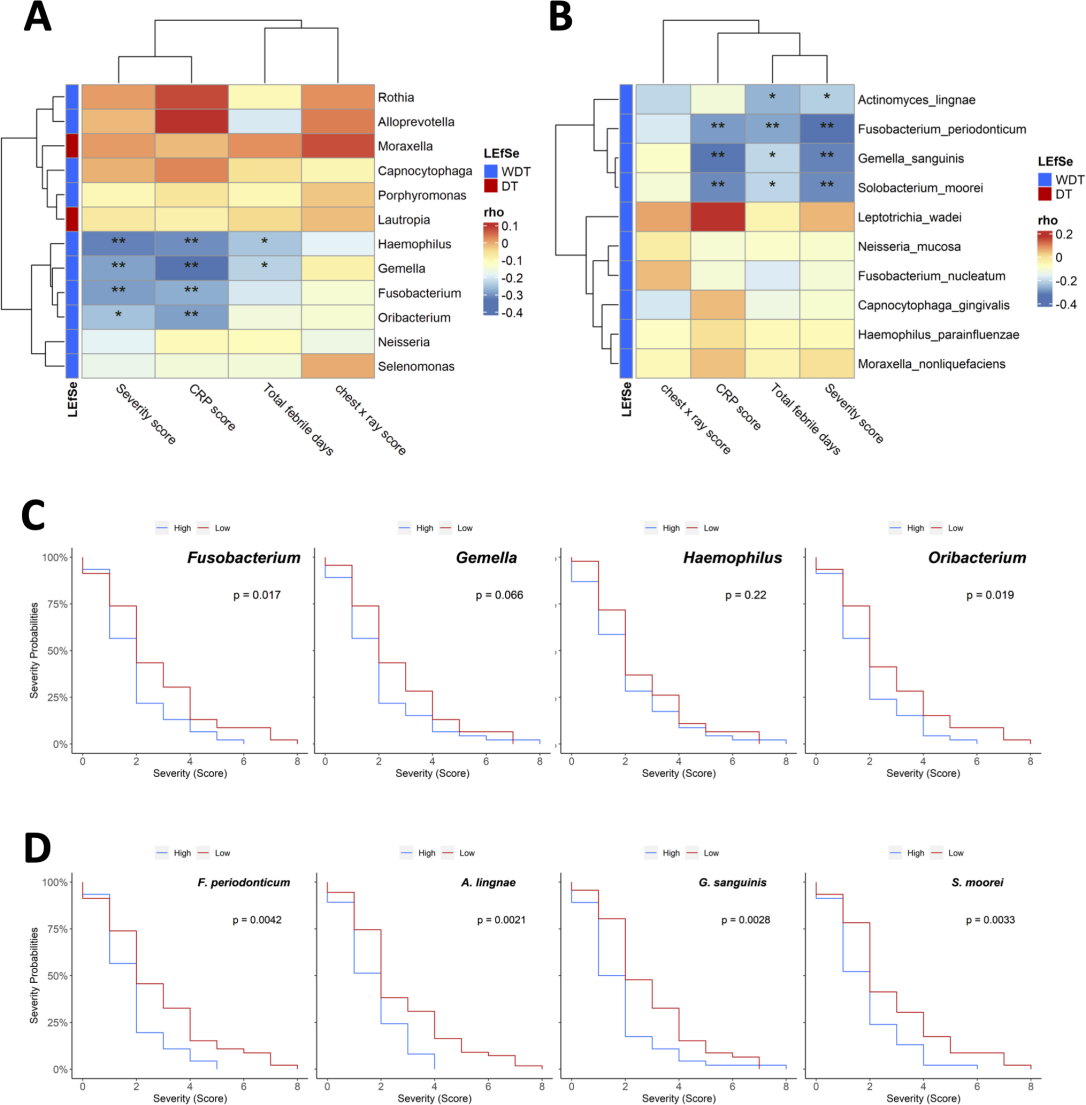
The association between the composition of the respiratory microbiota and clinical features of *Mycoplasma pneumoniae* infection. Pairwise Spearman rank correlation heatmap of significantly different taxa in throat swabs at initial sampling and clinical severity score WDT (blue) vs. DT (red) at the **(A)** genus and **(B)** species levels. Genus/species were in rows; the correlation was indicated by color gradient. **(C)** Comparison Kaplan–Meier plot severity score with high or low abundance of *Fusobacterium, Gemella, Haemophilus*, and *Oribacterium*. **(D)** Comparison Kaplan–Meier plot severity score with high or low abundance of *Fusobacterium periodonticum, Actinomyces lingnae, Gemella sanguinis*, and *Solobacterium moorei*. *p < 0.05, **p < 0.01.

Whether clinical disease severity was correlated with the respiratory bacterial levels in MRMP-infected patients with/without doxycycline treatment was examined by Spearman’s correlation analysis. Among the most relatively abundant genera in MRMP-infected patients without doxycycline treatment, *Fusobacterium*, *Haemophilus*, *Gemella*, and *Oribacterium* were inversely correlated with CRP and severity scores (**Figure 3A**). *Gemella* and *Haemophilus* were further negatively correlated with total febrile days. Among the most relatively abundant species in MRMP-infected patients without doxycycline treatment, *A. lingnae*, *F. periodonticum*, *G. sanguinis*, and *S. moorei* were negatively correlated with total febrile days and severity score (**Figure 3B**). *F. periodonticum*, *G. sanguinis*, and *S. moorei* were further negatively correlated with CRP. We divided patients into high versus low groups based on the median relative abundance of respiratory bacterial taxa to identify the specific microbial signature influencing treatment response in MRMP-mediated pneumonia patients. Patients with high *Fusobacterium* and *Oribacterium* abundance had lower severity scores than those with low abundance (*p* < 0.05) (**Figure 3C**). The relative abundance of these four species (*F. periodonticum*, *A. lingnae, G. sanguinis*, and *S. moorei*) were also significantly related to the severity score (**Figure 3D**). Because there is no difference in bacteria loads among patients, we proposed that microorganism-related metabolites might be related to later persistent symptoms, rather than the possibility of virulent ability and expansion of MRMP restricted by microorganisms.

### Changes in the respiratory metabolomic features between two groups

**Figure 4:**
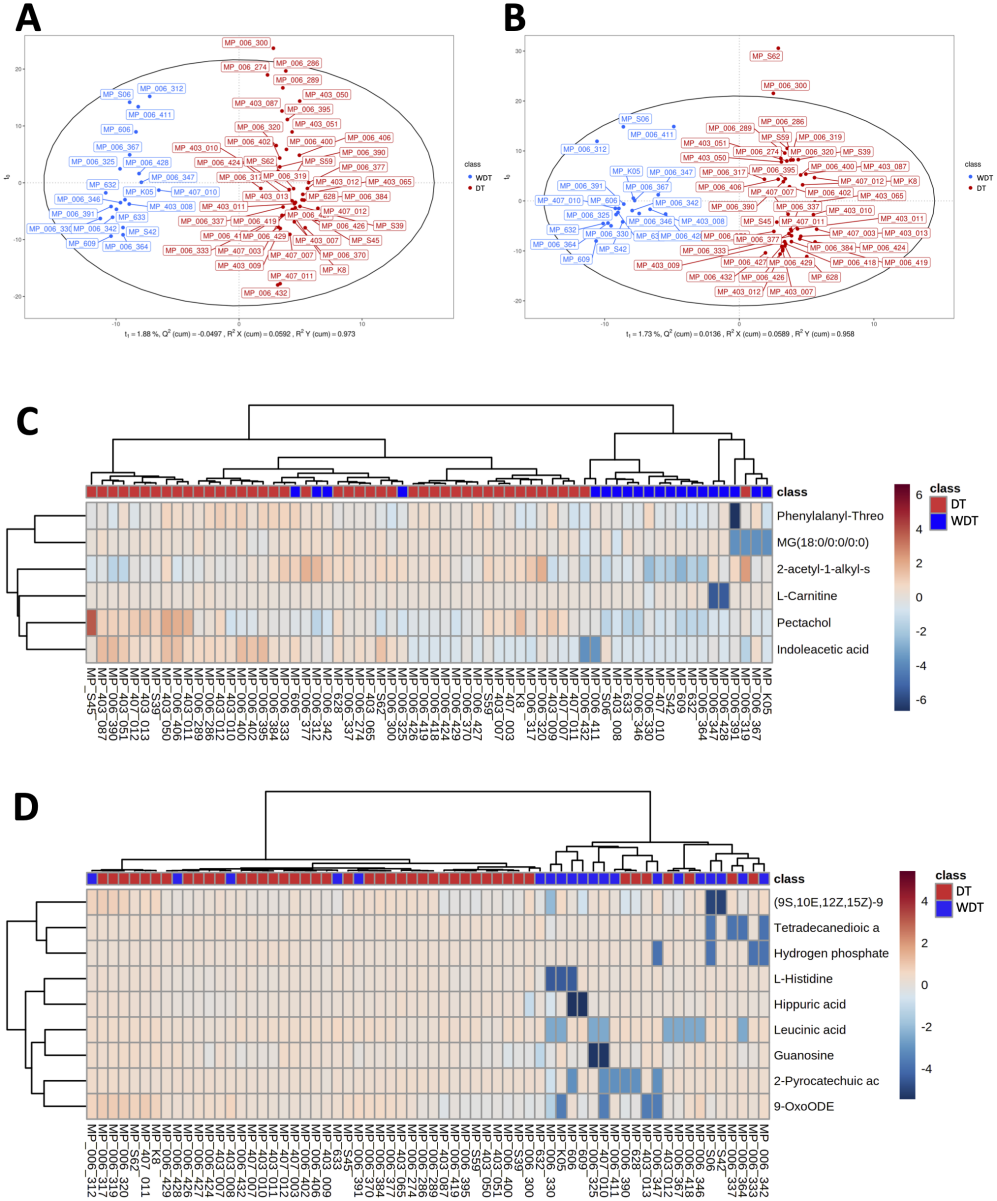
Comparison of respiratory metabolic profiles. Using orthogonal partial least squares discriminant analysis (OPLS-DA) score plot under **(A)** POS and **(B)** NEG modes ensured the detection stability of metabolomics data analysis. A bidirectional clustering analysis heatmap was used to visualize metabolite levels within two groups under **(C)** positive and **(D)** negative modes. The horizontal and vertical axes represented the samples and metabolites, respectively. Potential lipids were in rows and arranged according to hierarchical clustering by using Euclidean distance measure and Ward algorithm.

Human and bacterial metabolites play a critical role in modulating the immune system and significantly impact human health. To investigate these effects of MRMP infection, we conducted untargeted LC-MS/MS analysis to quantify the abundance of metabolites from MRMP-infected patients. OPLS-DA revealed clearly distinct metabolic profiles between WDT and DT patients under both positive and negative ionization modes (**Figures 4A, B**, respectively). From approximately 130 detected compounds, we identified 15 significantly altered metabolites (6 in positive and 9 in negative mode) associated with metabolic pathways, including phenylalanine, histidine, beta-alanine, tryptophan, and purine metabolism; lysine degradation and aminoacyl-tRNA biosynthesis; as well as amino acid and fatty acid metabolites. Bidirectional clustering heatmaps were generated to visualize these differences in patients with MRMP (**Figures 4C, D**).

### Microbial association with metabolites in *M. pneumoniae*-infected patients

**Figure 5:**
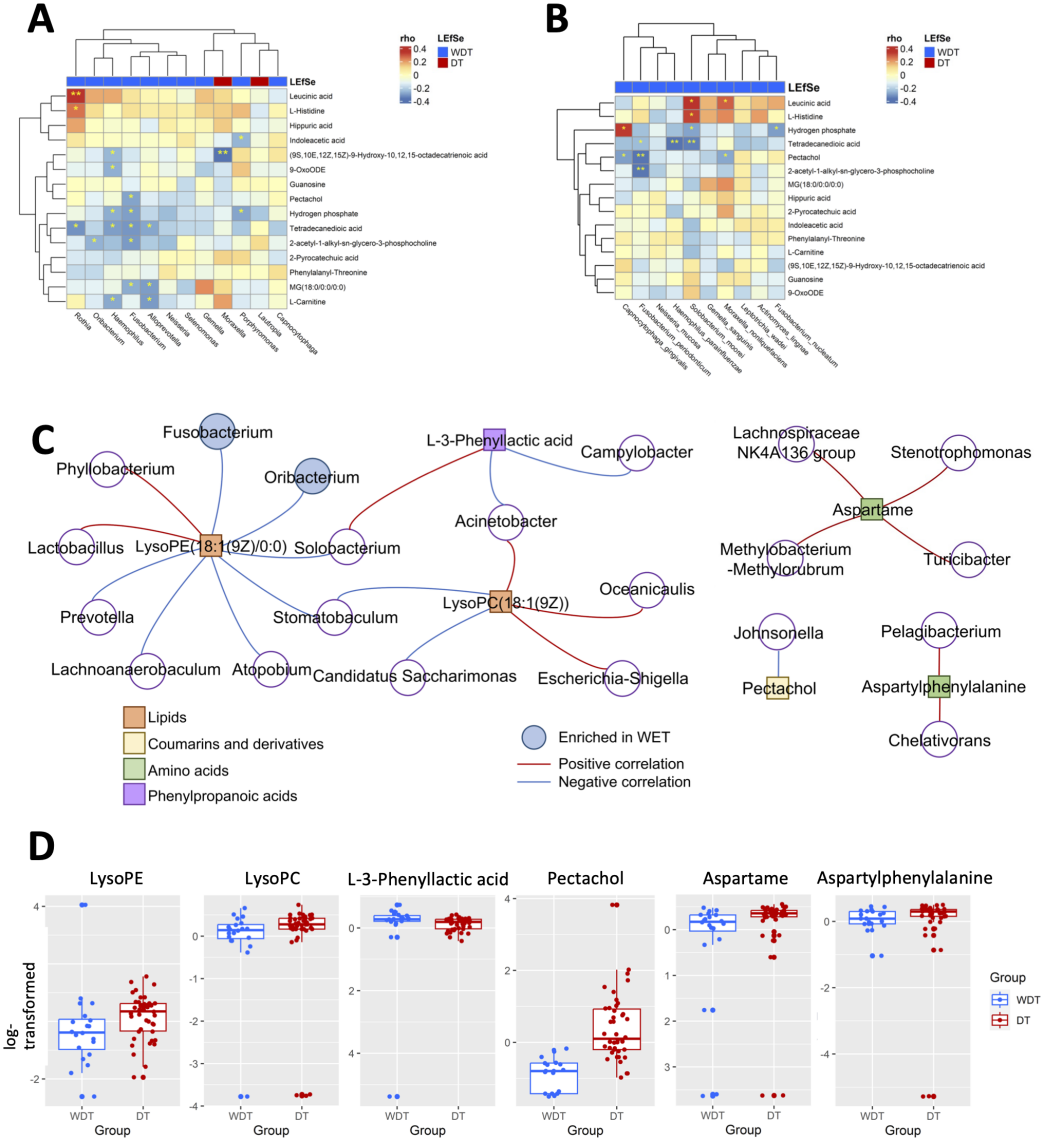
Integrated correlation-based network analysis of microbes and metabolites. Spearman’s correlation analysis was conducted using potential microbiome biomarkers of MRMP as identified by LEfSe and potential metabolite biomarkers identified through orthogonal projection to OPLS-DA at the **(A)** genus and **(B)** species levels. **(C)** The cluster networks of Spearman’s correlation using microbiome and metabolomics data. **(D)** The abundance of LysoPE, LysoPC, L-3-phenyllactic acid, pectachol, aspartame, and aspartylphenylalanine was analyzed (Wilcoxon rank sum test; *p < 0.05, **p < 0.01, ***p < 0.001).

To investigate the correlation between 15 significantly changed metabolites and 12 significantly different genera, a correlation matrix was generated by calculating the Spearman’s correlation coefficient (**Figure 5A**). *Moraxella* enriched in DT patients have some strong negative correlations with (9S,10E,12Z,15Z)-9-hydroxy-10,12,15-octadecatrienoic acid. The genera enriched in WDT patients have moderately negative correlations with 11 changed metabolites. However, *F. periodonticum* was significantly inversely correlated with pectachol and 2-acetyl-1-alkyl-sn-glycero-3-phosphocholine (**Figure 5B**).

### A disease-associated network in children with MRMP-mediated pneumonia

Based on these respiratory microbiome and metabolomics data, potential microbe-associated metabolites in *M. pneumoniae*-infected patients were identified using Spearman’s correlation-based analysis. Interestingly, we observed several metabolite–microbe correlation clusters (**Figure 5C**). The first network consisted of both LysoPE(18:1(9Z)/0:0) and LysoPC(18:1(9Z)), which was significantly decreased in WDT patients (**Figure 5D**) and was correlated with 14 genera (**Figure 5C**). Two of the 14 genera, *Fusobacterium* and *Oribacterium*, were enriched in WDT patients. A previous study indicated that LysoPE and LysoPC were glycerophospholipids, which mediated airway inflammation and were involved in signaling and immune responses through producing lysophospholipids (Gai et al., 2021). Although pectachol and aspartylphenylalanine networks were significantly increased in DT patients, they were not composed of any genus enriched in WDT and DT patients.

## Discussion

### Key findings

This study revealed that respiratory microbiome in patients suffering from MRMP infection was strongly related to the need for doxycycline treatment. Our results demonstrated that specific bacterial taxa may affect disease severity and the need for doxycycline treatment.

### Microbiota variance

The upper respiratory tract of healthy humans is inhabited by a substantial mass of bacterial communities and the oropharynx has the highest microbiota density (Bassis et al., 2015). Respiratory microbiome establishment and development are affected by several factors, such as delivery type, diet, number of siblings, daycare attendance, and antibiotic usage. They closely regulate host growth, shape immune maturation, and modify susceptibility to pathogenic microorganisms (Abt et al., 2012; Bassis et al., 2015). These commensal and symbiotic microbial communities can directly influence pathogens’ growth by antimicrobial production or nutrient/adhesion competition, and modulate innate and adaptive immunity responses, such as maintaining interferon expressions in the steady state (Abt et al., 2012) or increasing GM-CSF production in response to pulmonary bacterial infections (Brown et al., 2017). The composition of airway microbiota also influences the predisposition of respiratory diseases. For example, *Staphylococcus aureus* is negatively associated with respiratory syncytial virus (RSV) infection, but *Haemophilus influenzae* and *Streptococcus* spp. increased the vulnerability to RSV infection (de Steenhuijsen Piters et al., 2016). Other studies demonstrate that *Streptococcus* spp. colonization inhibits influenza replication, being less predisposed to influenza infection (Tsang et al., 2020). In contrast, *Prevotella* spp. is associated with increased influenza infection susceptibility and severity (Tsang et al., 2020). In this study, we expanded our sample by collecting data from the northern and southern regions of Taiwan county. Our result attempted to minimize the effect of community, lifestyle, and trained immunity backgrounds. We found limited diagnostic differences among patients other than with/without doxycycline and febrile days. We proposed that microbiota and related metabolite variances might be a major cause of the extended therapeutic process.

We demonstrated that WDT patients had higher microbial richness and diversity with a greater proportion of *Fusobacteria*, *Haemophilus*, *Gemella*, *Oribacterium*, *A. lingnae*, *F. periodonticum*, *G. sanguinis*, and *S. moorei*, which were associated with decreasing disease severity. Although *F. periodonticum*, *G. sanguinis*, and *S. moorei* cause periodontal disease and oral gastrointestinal cancer (Manson McGuire et al., 2014), they are also ubiquitous elements of healthy oral cavity (Yoneda et al., 2014). Studies show that oral *Fusobacteria* decorate their surface with sialic acid either for evasion from host immune system or for regulating host immune response by interacting with sialic acid-binding lectin (Lamprinaki et al., 2021). Meanwhile, sialic acid as a terminal residue on respiratory glycoconjugates is a primary target for *M. pneumoniae* attachment and gliding on airway epithelium, which is involved in *M. pneumoniae*-mediated pathogenesis and infectious processes (Williams et al., 2018). Recent studies have also shown that *F. periodonticum* is negatively correlated with the severity of patients with COVID-19 infection possibly through interaction with sialic acid, which mediates SARS-CoV-2 binding and viral entry (Nardelli et al., 2021).

### Metabolome changes might mediate disease development

Intriguingly, our study indicated that *Fusobacteria* was further inversely correlated with glycerophospholipid-related products: LysoPC, LysoPE, and 2-acetyl-1-alkyl-sn-glycero-3-phosphocholine. According to KEGG glycerophospholid metabolism, *F. periodonticum* could play a role in phosphotidylcholine (PC), phosphotidylethanolamine (PE), and sn-glycero-3-phosphocholine metabolism (**Supplementary Figure S3**) (KEGG Fusobacterium periodonticum T06291: C4N17_07735, fpei00564 Glycerophospholipid metabolism). In general, the glycerophospholipid family, representing 80% of cell membrane lipid, contains phosphatidic acid (PA), PC, and PE (Yetukuri et al., 2007). A previous study indicated that activation of innate immune cells stimulates phospholipase A2 to convert PC and PE into LysoPC and LysoPE by removing one of their fatty acid groups (O'Donnell et al., 2018). The biological functions of LysoPC and LysoPE are involved in proinflammatory activities, adhesion molecule upregulation, neutrophil activation, and chemotactic migration (Yoder et al., 2014). In addition, 2-acetyl-1-alkyl-sn-glycero-3-phosphocholine is a platelet-activating factor (PAF) and an inflammatory mediator secreted by many immune cell types, endothelial cells, and platelets, which contributes to leukocyte chemotaxis and local inflammation in response to bacterial and viral infection (Kelesidis et al., 2015). Taken together, it would be interesting to investigate whether *Fusobacteria* would be involved in host glycerophospholipid metabolism for self-consumption and prevent exaggerated inflammation response in the future. Importantly, our findings demonstrated that the participation of increasing lipid metabolism in patients with MRMP who needed doxycycline treatment is consistent with a previous study. In a previous study, dysregulated glycerophospholipid metabolism in the plasma of patients with *M. pneumoniae* pneumonia reflected cell membrane damage, immunity activation, along with hyperinflammation and underlying *M. pneumoniae* infection pathogenesis (Li et al., 2022). Our study suggests that oral *Fusobacteria*, together with other microbial species, may work to competitively utilize host-derived sialic acid with *M. pneumoniae*, hence providing a steady metabolite microenvironment to promote immune tolerance. Thus, decreasing inflammatory metabolites sustain homeostasis and further lead to a self-limited disease course, which results in patients who are enriched in microbial species, especially oral *Fusobacteria*, not necessarily requiring doxycycline treatment.

### Strengths and limitations

Our study might have several limitations. First, we used 16S rRNA gene sequencing for bacteria profiling because acquiring large amounts of concentrated DNA from the oropharyngeal swabs is difficult, which may restrict lower taxonomical resolution compared with whole shotgun metagenomic sequencing. Second, only the metabolome in oropharyngeal swab was analyzed, and this content might not be sufficient to comprehensively mirror the whole systemic picture. Nevertheless, our results demonstrated that a complex interplay between *M. pneumoniae* and respiratory microbiota during disease development is associated with dysregulated local metabolome, thus affecting disease severity and response to antibiotic treatment. Third, to determine whether certain distinct bacterial taxa including *F. periodonticum* would affect host immune response modulation and decrease disease severity, detailed mechanistic studies need to be conducted. These findings open new perspectives and strategies for diagnostic biomarkers of and therapeutic approaches to *M. pneumoniae*-mediated diseases. Further large-scale validation studies for clinical applications, based on these differential microbial and metabolic signatures, hold significant potential as indicators for assessing disease progression and treatment response and for identifying novel targets for therapeutic interventions.

## Data Availability

The 16S sequence reads presented in the study have been deposited in NCBI BioProject under the accession number PRJNA954547.
