# Supplementary material for: Respiratory microbiome and metabolome features associate disease severity and the need for doxycycline treatment in children with macrolide-resistant Mycoplasma pneumoniae-mediated pneumonia
Source: Front Cell Infect Microbiol. 2025 Jul 28;15:1537182. doi: 10.3389/fcimb.2025.1537182 (PMC12336209; doi:10.3389/fcimb.2025.1537182)
Supplement: Supplementary file 1 [file Table1.docx]

**Supplemental Appendix**

**Respiratory microbiome and metabolome features associate disease severity and the need of doxycycline treatment in children with macrolide-resistant *Mycoplasma pneumoniae*-mediated pneumonia**

**Supplementary figures**

**Supplementary figure 1**

**
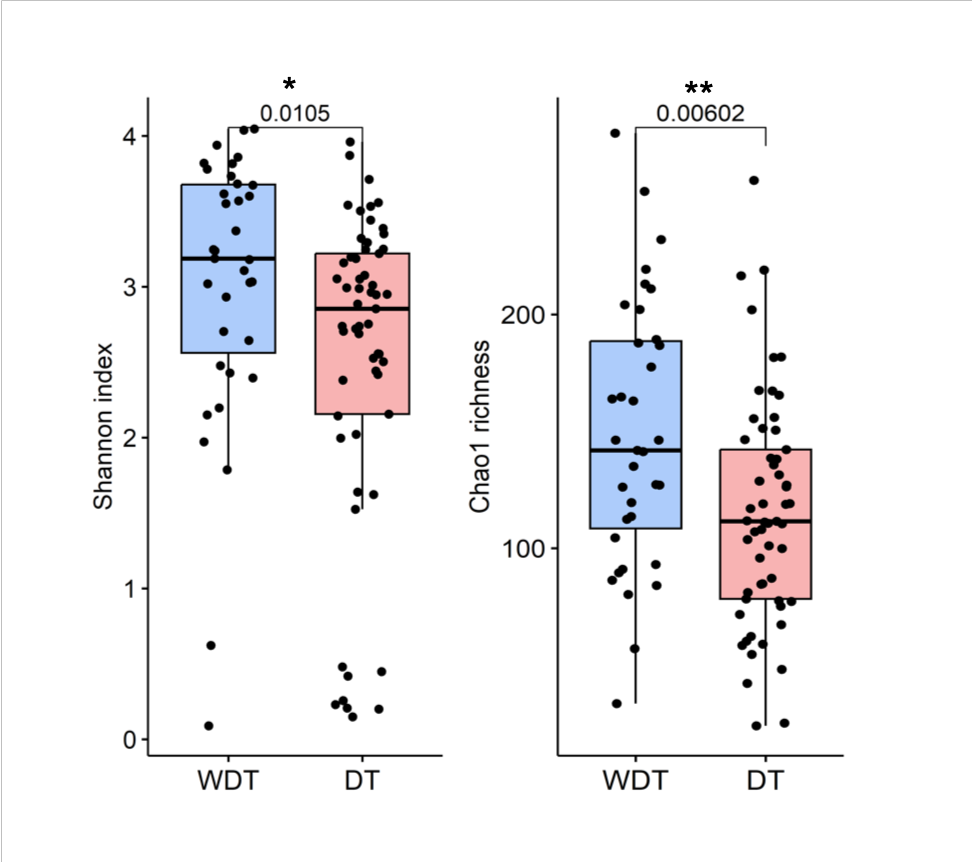
**

**Supplementary figure 2**

**
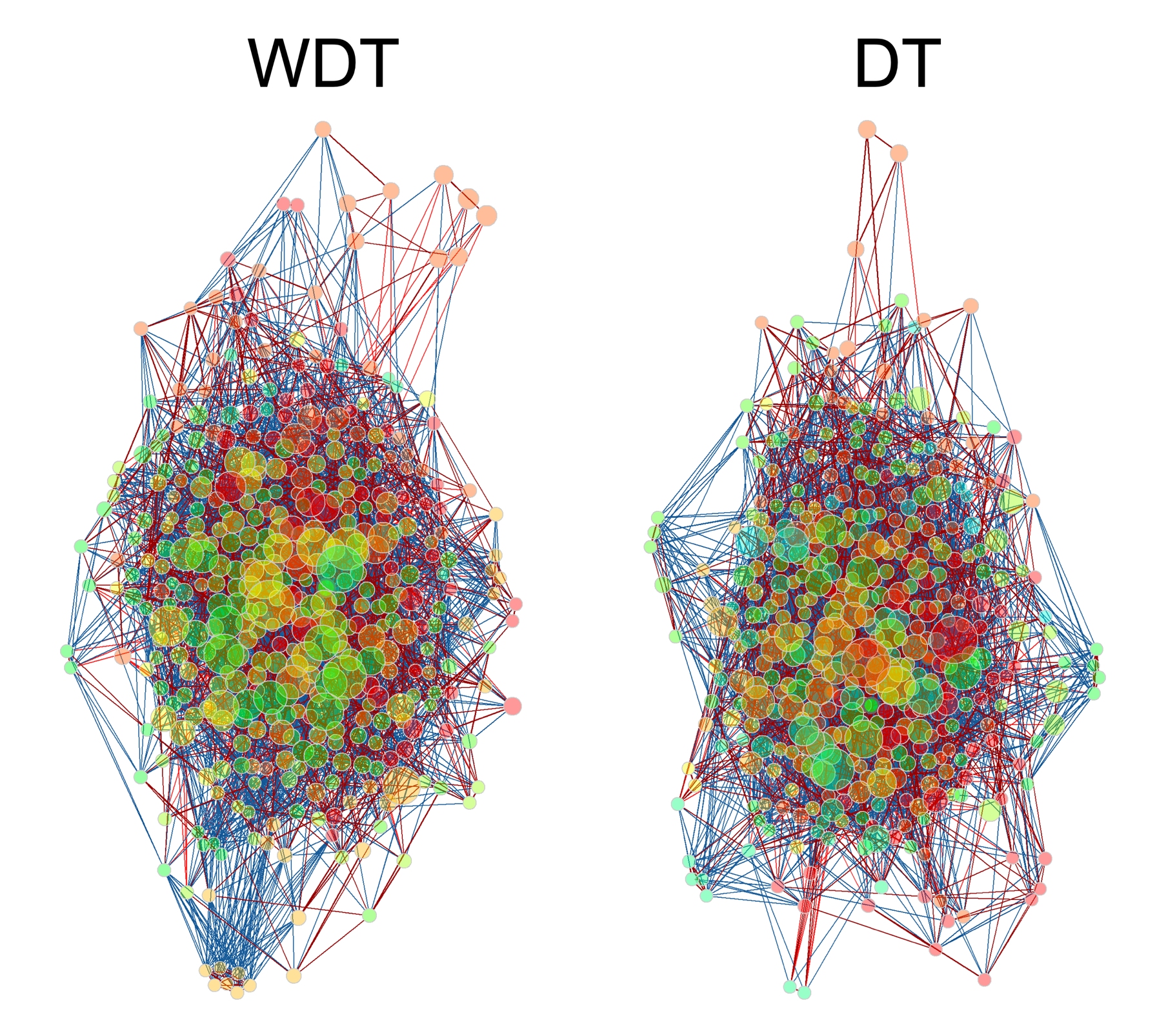
**

**Supplementary figure 3**

**
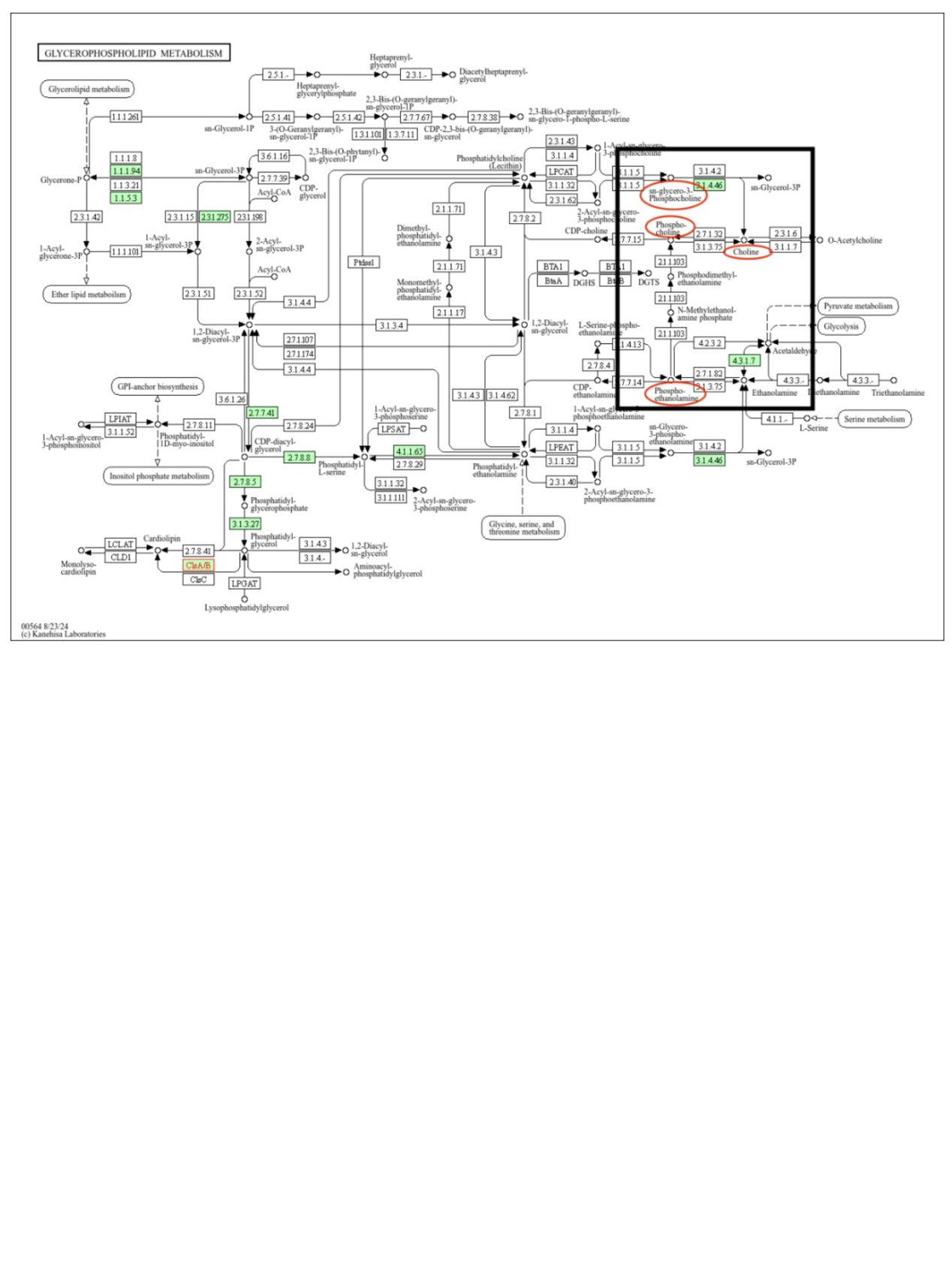
**

**Supplementary figure 4**

**Supplementary figure legends**

**Supplementary figure 1. WDT and DT presented different microbiome diversities.**

Chao1 richness and Shannon-diversity index were conducted to assess microbiome diversity in throat swabs samples. Significant differences between the WDT (blue) and DT (red) groups were evaluated by Kruskal-Wallis test. * *P*<0.05. ***P*<0.01.

**Supplementary figure 2. WDT and DT presented different networks distributions.**

Comparison of networks were generated from the WDT (left) and DT (right) samples of the throat swabs data set using setting and parameters. The size of the nodes was adjusted by a normalization of the counts, and a color was assigned to each subcluster within their corresponding networks. Positive associations were shown with green connections and negative correlations with red connections.

**Supplementary figure 3. KEGG glycerophospholipid metabolism pathway of *Fusobacteria periodonticum.***Green icons present *F. Periodonticum* related metabolites involved in Phosphocholine (PC), Phosphotidylethanolamine (PE) and sn-glycero-3-phosphocholine metabolism.

**Supplementary figure 4. Volcano plots of differential metabolites of WDT and DT groups.** (A) Volcano plot in positive ion mode. (B) Volcano plot in negative ion mode. The x-axis represents the log2 fold change (log2 FC), and the y-axis represents the -log10 p-value derived from Student’s t-test. Metabolites are color-coded based on their regulation status: red dots indicate significantly upregulated metabolites (log2 FC > 1, p < 0.05), blue dots indicate significantly downregulated metabolites (log2 FC < -1, p < 0.05), and gray dots represent metabolites with no significant change. VIP, variable importance in projection from the OPLS-DA model.

**Supplementary material and methods**

**Patients, sample collection, and ethical statement**

Chang Gung Memorial Hospital Lin-Kou branch (LCGMH) is a 3,700-bed medical centre, which provides both primary and tertiary care in northern Taiwan. Saint Paul’s Hospital is a 450-bed regional hospital, which provides primary and secondary care in northern Taiwan. CGMH-Kaohsiung branch (KCGMH), a 2,700-bed medical centre in southern Taiwan joined into this study from January 2019. After written informed consents were obtained from patients or their parents, throat swabs were collected from the patients using a sterile swab (FLOQSwabs, Copa; Murrieta, CA, USA) by paediatricians within 48h after admission. This study protocol was approved by the research ethics committee of the Chang Gung Memorial Hospital (CGMH), Taiwan (Approval number: 201900420A3 and 202000687B0). All throat swabs were sent to the laboratory at LCGMH.

**16S rRNA-targeted library preparation and sequencing**

PCR and sequencing were performed as described in the 16S metagenomics Sequencing Library preparation protocol of Illumina. Genomic DNA was subjected to amplification targeting the V3-V4 hypervariable regions of the bacterial 16S rRNA gene using region-specific primers with Illumina overhang adapter sequences. The forward primer sequence was 5’-TCGTCGGCAGCGTCAGATGTGTATAAGAGACAGCCTACGGGNGGCWGCAG-3’ and the reverse primer sequence was 5’-GTCTCGTGGGCTCGGAGATGTGTATAAGAGACAGGACTACHVGGGTATCTAATCC-3’, where the underlined portions correspond to the Illumina Nextera XT adapter sequences required for subsequent indexing. PCR amplicons were purified with AMPure XP magnetic beads (Beckman Coulter). A secondary PCR was conducted to attach dual indices and sequencing adapters using the Nextera XT Index Kit (Illumina), after which the indexed libraries were purified again using AMPure XP beads. The concentration and quality of the libraries were evaluated using a Qubit Fluorometer (Thermo Fisher Scientific) and an Agilent Bioanalyzer (Agilent Technologies). Equimolar pooling of purified libraries was performed prior to sequencing. Sequencing was carried out on the Illumina MiSeq platform.

**16S metagenomic bioinformatics analysis**

16S rRNA gene sequences were processed to classify microbial constituents using the DADA2 pipeline ^1^ for modelling and amplicon error correction, followed by quality trimming, denoising, merging, and chimaera removal. The truncated forward and reverse sequences were defined at positions 290 and 220, respectively, and the first 13 bases of each sequence were trimmed. Total sequences were used to construct amplicon sequence variants (ASVs), and ASVs comprising <10 reads in at least two samples were filtered from the dataset. A Naïve Bayes classifier was trained by using the most recent available version of Silva (release 138.1) sequences for taxonomy assignment. We used standard filtering parameters to process the raw reads and remove potential human DNA contamination. Sequence reads were classified at several taxonomic levels: kingdom, phylum, class, order, family, genus, and species.

**Untargeted metabolomic profiling and analysis in human throat (respiratory tract)**

The untargeted metabolomic profiling of human throat samples was performed by BIOTOOLS CO., LTD. (Taiwan). All samples were stored at –80 °C until assayed. Before the analysis, the samples were thawed at 4 °C on ice. Raw data files were converted into the mzML format by using ProteoWizard and processed by using the R package XCMS (version 3.2). Pre-processed data were normalised by sum of total peak area, log transformed, and autoscaled (mean-centred and divided by the standard deviation of each variable) prior to downstream statistical analysis. For multivariate statistical analysis, orthogonal projection to latent structures-discriminant analysis (OPLS-DA) was performed by using Metaboanalyst 5.0 as in previous study ^2^. The predictive performance of the PLS-DA model was assessed by using the cumulative Q2Y metric, which had values between 0 and 1. High Q2Y indicated excellent performance. Differential metabolites were filtered by using the variable importance in projection (VIP) score generated from the OPLS model and the *P-*value obtained from Student’s *t*-test. The metabolites were statistically significant if VIP ≥1.5 and *P* <0.05. For univariate analysis, significant metabolites differentially regulated in the two groups were analysed with volcano plots that included |log_2_F.C.| <1 and *P* <0.05 of Student’s *t*-test.

**Supplementary reference**

1. Callahan BJ, McMurdie PJ, Rosen MJ, Han AW, Johnson AJA, Holmes SP. DADA2: High-resolution sample inference from Illumina amplicon data. *Nature Methods* 2016;**13**(7):581-3.

2. Pang Z, Chong J, Zhou G, de Lima Morais DA, Chang L, Barrette M, et al. MetaboAnalyst 5.0: narrowing the gap between raw spectra and functional insights. *Nucleic Acids Research* 2021;**49**(W1):W388-W96.
